# Clinical efficacy of optical coherence tomography parameters to predict the visual field outcome following pituitary adenoma surgery

**DOI:** 10.1371/journal.pone.0313521

**Published:** 2024-11-12

**Authors:** Kwang Eon Han, Heeyoung Choi, Su-Jin Kim, Seung Min Lee, Ji-Eun Lee

**Affiliations:** 1 Department of Ophthalmology, Research Institute for Convergence of Biomediocal Science and Technology, Pusan National University Yangsan Hospital, Pusan National University School of Medicine, Pusan National University Yangsan Hospital, Yangsan, Korea; 2 Department of Ophthalmology, Biomedical Research Institute, School of Medicine, Pusan National University, Pusan National University Hospital, Busan, Korea; Hangil Eye Hospital / Catholic Kwandong University College of Medicine, REPUBLIC OF KOREA

## Abstract

**Purpose:**

To investigate the factors affecting visual field recovery in patients with pituitary adenoma following surgical removal, both eyes of 35 patients with pituitary adenoma who had been followed up for > six months post-surgery were retrospectively analyzed.

**Material and methods:**

Pre- and post-operative visual acuity, visual field test, retinal nerve fiber layer (RNFL), and ganglion cell inner plexiform layer (GCIPL) thickness were investigated. The average age of the 35 patients was 58.3 ± 11.5 years. Preoperatively, 30 eyes (mean average RNFL thickness, 99.73 ± 5.89 μm) and 40 eyes (mean average RNFL thickness, 77.55 ± 8.35 μm) were included in the thick (≥ 90 μm) and thin RNFL group (< 90 μm), respectively.

**Results:**

In the thick RNFL group, pre- and post-operative mean deviation (MD) and pattern standard deviation (PSD) were favorable (all p < 0.001), and the proportion of eyes of postoperative MD change which were stable or improved was greater than in the thin RNFL group (p = 0.042). Preoperative MD, RNFL (except nasal quadrant) and GCIPL thickness were positively correlated to postoperative MD values (all, p < 0.05). Preoperative MD and temporal RNFL thickness were significantly correlated with postoperative MD change rate (p = 0.03 and 0.04, respectively). Preoperative GC IPL thickness and postoperative MD change rate were not significantly correlated (p = 0.61). Using univariate regression analysis, preoperative best corrected visual acuity (Odds ratio [OR], 0.050; p *<* 0.001), tumor volume (OR, 1.110, p = 0.002), higher preoperative MD values (OR, 0.858; p < 0.001), lower preoperative PSD values (OR, 1.169, p = 0.002), thick RNFL (OR, 0.215; p = 0.003) and thick GCIPL (OR, 0.305, p = 0.018) were significantly associated with a good visual field outcome following surgery. According to multivariate analysis, any other parameters were not significant. In patients with thick RNFL, postoperative MD values were better than in the thin RNFL group.

**Conclusions:**

Eyes with preoperative higher MD and thick temporal RNFL showed more improvement in their visual fields postoperative. Preoperative thick RNFL could be a potential predictor of visual field recovery following TSA-TR, while macular GCIPL thickness does not appear to be a reliable predictor.

## Background

Pituitary tumors expand upward and compress the crossed fibers in the optic chiasm, which affects the nasal hemiretina and leads to visual field (VF) defects, typically bitemporal hemianopia, decreased visual acuity (VA), or color perception [1]. Prolonged chiasmal compression can result in band atrophy of the temporal and nasal sectors of the optic disc. While surgical removal of pituitary adenomas is generally successful, complete restoration of visual function may not be achieved in some patients [2–7].

Numerous researchers have investigated preoperative prognostic factors for visual function improvement. Optical coherence tomography (OCT) can detect retinal nerve fiber layer (RNFL) loss in eyes with chiasmal compression [8–11], and RNFL thickness has been found to predict visual outcomes in cases of pituitary tumors [12–14]. In a previous study, we reported that preoperative mean deviation (MD), temporal RNFL thickness, and symptom duration are predictive variables that affect postoperative visual field recovery [15]. Recently, the thickness of the ganglion cell-inner plexiform layer (GC IPL) has been identified as a detectable marker in the early or subclinical stages of various optic neuropathies [16,17]. Changes in macular GCIPL thickness precede changes in peripapillary RNFL thickness [18]. Some studies have suggested that GCIPL thickness could serve as a reliable predictor of central visual field impairment in patients with pituitary tumors [19].

However, there is a lack of comprehensive research on the correlation between RNFL, GCIPL, and VF test results before and after surgery, as well as prognostic factors that influence visual recovery. Therefore, the present study aims to evaluate the clinical efficacy of the visual field test, RNFL, and GCIPL thickness in predicting postoperative outcomes of visual fields in patients with pituitary adenoma.

## Materials and methods

We conducted a retrospective analysis of the medical records of 35 patients who underwent transsphenoidal approach-tumor resection (TSA-TR) for diagnosed pituitary adenoma at Yangsan Pusan National University Hospital between January 2020 and December 2022. The study adhered to the principles outlined in the Declaration of Helsinki, and it was approved by the institutional review board (IRB number: 55-2024-026). Since this study was retrospective in nature, informed consent was waived. For the purposes of this research, the data was accessed by the researchers between March 20, 2024, and April 10, 2024.

To be included in the study, patients had to have pituitary tumors confirmed to be compressing the anterior visual pathway through magnetic resonance imaging (MRI) and have undergone surgery. Only patients who had a postoperative follow-up of at least six months and had undergone preoperative assessments of visual acuity, dilated fundus examination, automated perimetry using a VF analyzer (Humphrey Field Analyzer; Carl Zeiss-Meditec, Dublin, CA, USA), and Cirrus HD OCT® (Carl Zeiss Meditec, Oberkochen, Germany) of the peripapillary RNFL and macular GC IPL) were included in the analysis. Patients with a history of treatments such as radiotherapy or chemotherapy, any ophthalmological abnormalities other than optic nerve disease or glaucoma, or any history of neurological disease leading to a VF defect were excluded. Only reliable data from the VF test results, which included a false positive error rate of less than 20%, false negative error rate of less than 20%, and fixation loss rate of less than 20%, were considered for analysis.

A visual field defect was defined as an MD value of less than −5.0 dB. Postoperative VF status was categorized into three groups: not improved (preoperative MD < −5.0 dB to postoperative MD < −5.0 dB), stable (preoperative MD ≥ −5.0 dB to postoperative MD ≥ −5.0 dB), improved (preoperative MD < −5.0 dB to postoperative MD ≥ −5.0 dB), or aggravated (preoperative MD ≥ −5.0 dB to postoperative MD < −5.0 dB).

We analyzed the average peripapillary RNFL thickness and the thickness of each quadrant (superior, inferior, temporal, and nasal). Patients were divided into two groups based on the average peripapillary RNFL thickness: normal average pRNFL group (≥ 90 μm) and thin average pRNFL group (< 90 μm). At six months after surgery, we evaluated best-corrected visual acuity (BCVA), VF, RNFL thickness, and GCIPL thickness for each patient.

Statistical analysis was performed using SPSS for Windows version 23.0 (SPSS Inc, Chicago, IL, USA). Descriptive statistics, including age, sex, visual acuity (logarithm of the minimum angle of resolution [logMAR]), duration of neurological symptom onset to surgery, pattern of visual field defect, and the presence of a central visual field defect, were calculated for all patients. Patient characteristics were compared using t-tests or chi-squared tests. Pearson’s correlation coefficient was used to analyze the correlation between the visual field test and RNFL thickness. Univariate and multivariate analyses were conducted for each variable to investigate the factors associated with postoperative visual field recovery. Statistical significance was defined as a p-value < 0.05.

## Results

The mean age of the patients in the study was 58.3 ± 11.5 years, with an equal distribution of 15 men and 15 women. Preoperative BCVA was 0.24 log MAR for the entire cohort (Table 1). Preoperatively, 30 eyes (mean average RNFL thickness, 99.73 ±5.89 μm) were included in the thick RNFL group (≥ 90 μm) and 40 eyes (mean average RNFL thickness, 77.55 ± 8.35 μm) were included in the thin RNFL group (< 90 μm). No statistically significant differences was present in sex, duration of symptoms, or BCVA between the two groups. However, the thick RNFL group had younger patients, higher pre- and postoperative MD values, lower pre- and postoperative PSD values, and GC IPL thickness in all quadrants compared to the thin RNFL group (Table 2).

**Table 1:** Baseline characteristics of the patients.

Variable	Value
Eyes / Patients	70/35
Sex (male / female)	18/21
Age (years)	58.3 ± 11.5
BCVA (logMAR)	0.24 ± 0.34
Duration of symptoms (weeks)	17.4 ± 22.8
Tumor size (mm)	26.1 ± 9.2
Pathology	
Non-functioning PA	33 (84.6)
Functioning PA	6 (15.4)
Visual field test	
MD (dB)	-9.81 ± 9.69
PSD (dB)	7.7 ± 5.21
RNFL thickness (μm)	
Average	87.1 ± 13.3
Superior quadrant	109.43 ± 21.45
Temporal quadrant	65.26 ± 17.76
Inferior quadrant	110.33 ± 20.76
Nasal quadrant	63.29 ± 11.13
GC IPL thickness (μm)	
Average	74.3 ± 13.22
Superior quadrant	75.11 ± 14.97
Temporal quadrant	75.08 ± 14.1
Inferior quadrant	72.71 ± 14.05
Nasal quadrant	70.09 ± 15.25

Values are presented as mean ± standard deviation, number, or number (%) unless otherwise indicated.

BCVA = best-corrected visual acuity; logMAR = logarithm of the minimum angle of resolution; MD = mean deviation; PA = pituitary adenoma; PSD = pattern standard deviation; RNFL = retinal nerve fiber layer; VFD = visual field defect; VFI = visual field index.

**Table 2:** Preoperative characteristics of patient groups according to preoperative RNFL thickness on OCT.

Variable	thick RNFL (≥90um)	thin RNFL (<90um)	*p*-value
Eyes^†^	30	40	N/A
Sex (male / female)^†^	9/21	21/19	0.060
Age (years)*	53.47 ± 13.26	61.15 ± 8.94	0.004
BCVA (logMAR)*	0.23 ± 0.36	0.24 ± 0.34	0.46
Duration of symptoms (weeks)			
Tumor size (mm)	20.67 ± 6.32	29.8 ± 8.56	<0.001
Pathology^†^			
Non-functioning PA			
Functioning PA			
Preoperative Visual field test			
MD (dB)*	-4.95 ± 7.98	-12.66 ± 9.24	<0.001
PSD (dB)*	4.92 ± 3.94	9.88 ± 5.36	<0.001
RNFL thickness (μm)*			
Average	99.73 ± 5.89	77.55 ± 8.35	<0.001
Superior quadrant	126.87 ± 12.32	96.35 ± 17.09	<0.001
Temporal quadrant	77.03 ± 15.79	56.43 ± 13.63	<0.001
Inferior quadrant	127.67 ± 11.54	97.33 ± 16.09	<0.001
Nasal quadrant	67.30 ± 8.25	60.28 ± 12.11	0.008
GC IPL thickness (μm)*			
Average	81.93 ± 9.64	68.68 ± 12.75	<0.001
Superior quadrant	83.39 ± 8.82	69.00 ± 15.71	<0.001
Temporal quadrant	80.39 ± 10.52	71.16 ± 15.21	0.008
Inferior quadrant	79.14 ± 12.53	67.97 ± 13.33	<0.001
Nasal quadrant	80.86 ± 10.71	62.16 ± 13.12	<0.001
Postoperative BCVA	0.14 ± 0.34	0.25 ± 0.34	0.141
Postoperative Visual field test			
MD (dB)*	-1.06 ± 4.56	-9.64 ±9.17	<0.001
PSD (dB)*	2.63 ± 1.83	9.89 ± 5.71	<0.001

Values are presented as mean ± standard deviation, number, or number (%) unless otherwise indicated.

N/A = not applicable; BCVA = best-corrected visual acuity; logMAR = logarithm of the minimum angle of resolution; MD = mean deviation; PA = pituitary adenoma; PSD = pattern standard deviation; RNFL = retinal nerve fiber layer; VFD = visual field defect; VFI = visual field index.

*Comparison between groups by Student *t*-test.

^†^Comparison between groups by Pearson’s chi-square test.

**Fig 1:**
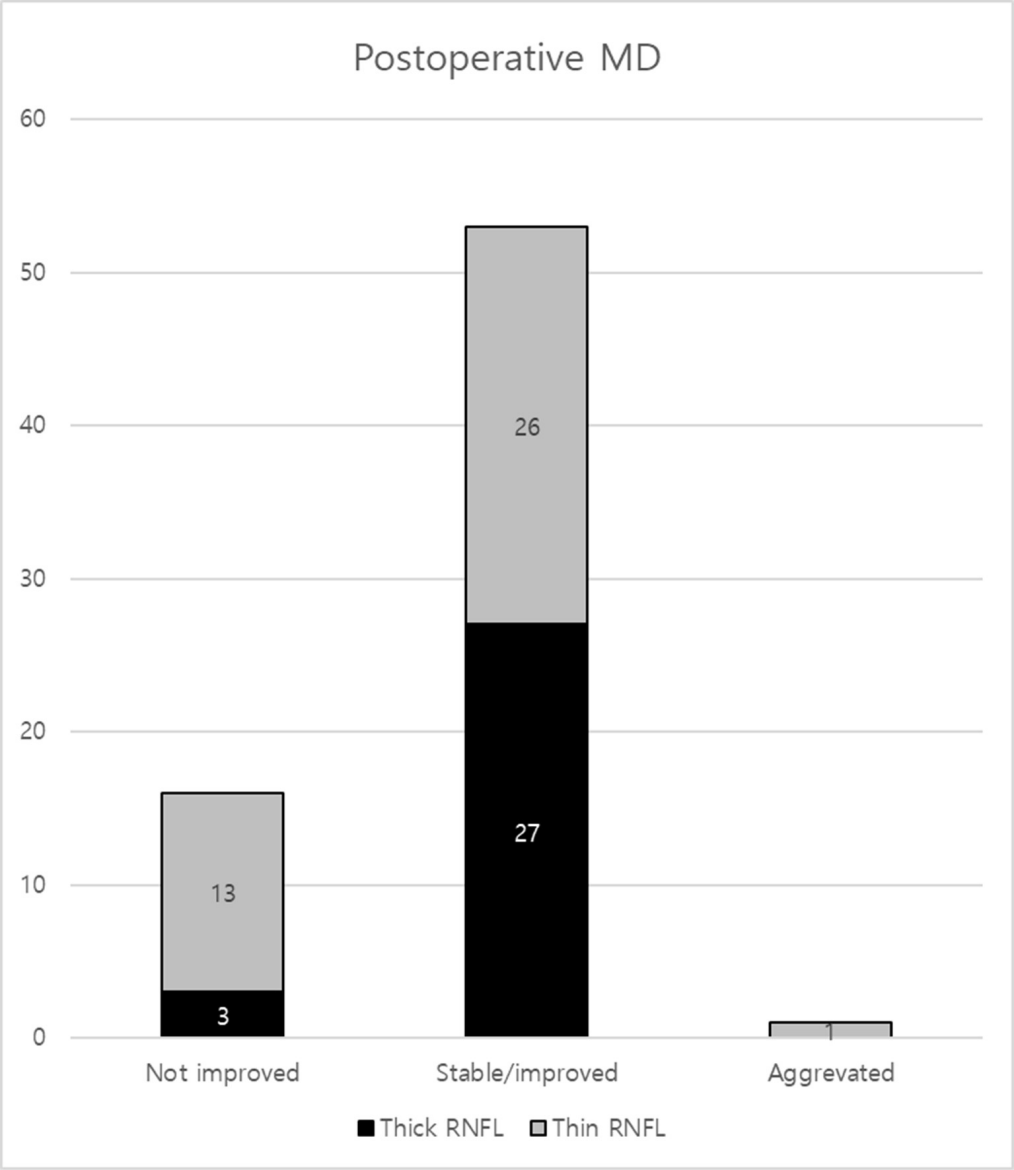
Graphs showing 3 categories (not improved, stable or improved, and aggravated) of postoperative visual field outcome in the thick and thin RNFL groups.

In the thick RNFL group, 26 eyes showed stable or improved postoperative MD values, while three eyes did not show improvement. In the thin RNFL group, 26 eyes showed stable or improved MD values, 13 eyes did not show improvement, and one eye’s MD worsened (p = 0.042) (Fig 1).

Preoperative MD, average RNFL thickness, superior RNFL thickness, temporal RNFL thickness, inferior RNFL thickness, and average GCIPL thickness in all quadrants were positively correlated with postoperative MD values. There was a significant positive correlation between preoperative temporal peripapillary RNFL thickness and the postoperative MD change rate (calculated as [postoperative MD—preoperative MD]/preoperative MD * 100, %) (p = 0.04). Additionally, there was a significant positive correlation between preoperative MD and the postoperative MD change rate (p = 0.03). However, there was no significant correlation between preoperative GC IPL thickness and the postoperative MD change rate (p = 0.61) (Table 3).

**Table 3:** Correlation between preoperative MD, PSD, RNFL, GC IPL thickness and postoperative MD change.

Variable	PostOp MD		MD change		MD Relative change	
	R	*p*-value*	R	*p*-value*	R	*p*-value*
Visual field test						
PreOp MD (dB)	0.743	<0.001	-0.596	<0.001	-0.296	0.03
PreOp PSD (dB)	-0.131	0.36	0.219	0.12	0.164	0.25
RNFL thickness (μm)						
Average	0.625	<0.001	-0.103	0.50	-0.27	0.07
Superior quadrant	0.636	<0.001	-0.179	0.24	-0.208	0.17
Temporal quadrant	0.391	0.008	-0.013	0.93	-0.291	0.04
Inferior quadrant	0.579	<0.001	-0.083	0.59	-0.19	0.21
Nasal quadrant	0.141	0.36	0.054	0.73	-0.133	0.38
GC IPL thickness (μm)						
Average	0.546	0.001	-0.081	0.61	-0.083	0.61
Superior quadrant	0.426	0.005	-0.149	0.35	-0.088	0.59
Temporal quadrant	0.672	<0.001	-0.057	0.72	-0.137	0.39
Inferior quadrant	0.515	<0.001	-0.044	0.79	-0.005	0.98
Nasal quadrant	0.513	<0.001	-0.049	0.76	-0.096	0.55

Values are presented as mean ± standard deviation, number, or number (%) unless otherwise indicated.

MD = mean deviation; PreOp = preoperative; PostOp = postoperavtive; PSD = pattern standard deviation; R = correlation coefficient, RNFL = retinal nerve fiber layer; VFI = visual field index.

Relative change = (preoperative value—postoperative value) / preoperative value (% presentation).

*Pearson correlation coefficient analysis.

Univariate analyses revealed that BCVA (odds ratio [OR], 0.050; p < 0.001), tumor volume (OR, 1.110; p = 0.002), higher preoperative MD values (OR, 0.858; p < 0.001), lower preoperative PSD values (OR, 1.169; p = 0.002), thick average RNFL (OR, 0.215; p = 0.003), and thick average GC IPL (OR, 0.305; p = 0.018) were associated with a favorable visual field outcome following surgery. However, in multivariate analyses, no significant factors were found to predict a favorable visual field outcome (Table 4).

**Table 4:** Preoperative factors influencing visual field improvement after surgery of pituitary tumors.

Variable	Univariate analysis			Multivariate analysis		
	OR	95% CI	*p*-value^*^	OR	95% CI	*p-value* ^*^
Age (years)	1.008	0.966–1.051	0.715			
Sex (male)	1.000	0.389–2.571	1.000			
BCVA (logMAR)	0.050	0.010–0.254	<0.001	0.114	0.009–1.439	0.093
Duration of symptoms (weeks)	0.975	0.930–1.021	0.283			
Tumor volume (cm^3^)	1.110	1.038–1.187	0.002	1.044	0.933–1.168	0.454
Pathology (Functioning)	1.000	0.171–5.851	1.000			
Visual field test						
MD (dB)	0.858	0.800–0.921	<0.001	0.931	0.826–1.050	0.243
PSD (dB)	1.169	1.058–1.291	0.002	1.184	0.999–1.403	0.052
RNFL thickness (μm)						
Average	0.934	0.893–0.977	0.003	1.061	0.914–1.233	0.437
Superior quadrant	0.951	0.922–0.980	0.001	0.977	0.906–1.053	0.541
Temporal quadrant	0.968	0.936–1.001	0.056			
Inferior quadrant	0.976	0.952–1.002	0.065			
Nasal quadrant	0.963	0.912–1.017	0.176			
GC IPL thickness (μm)						
Average	0.947	0.904–0.991	0.020	0.974	0.796–1.190	0.795
Superior quadrant	0.956	0.919–0.995	0.026	0.980	0.911–1.054	0.590
Temporal quadrant	0.965	0.927–1.005	0.088			
Inferior quadrant	0.958	0.917–1.000	0.052			
Nasal quadrant	0.940	0.901–0.981	0.004	1.011	0.933–1.095	0.793

Values are presented as mean ± standard deviation, number, or number (%) unless otherwise indicated.

BCVA = best-corrected visual acuity; CI = confidence interval; logMAR = logarithm of the minimum angle of resolution; MD = mean deviation; OR = odds ratio; PSD = pattern standard deviation; RNFL = retinal nerve fiber layer; VFD = visual field defect; VFI = visual field index.

*Logistic regression analysis.

## Discussion

In this study, the thickness of the preoperative peripapillary RNFL and macular GC IPL, both were evaluated as a predictor of postoperative visual field outcomes in patients who underwent transsphenoidal approach-tumor resection (TSA-TR). The results showed that patients with a thick RNFL had better postoperative visual field outcomes compared to those with a thin RNFL. Specifically, eyes with a thick temporal RNFL showed a greater improvement in MD in the visual field. The proportion of eyes with stable or improved postoperative MD values was also greater in the thick RNFL group compared to the thin RNFL group.

The recovery of VF defects and visual impairments following TSA-TR has been one of the most important issues regarding prognosis. Visual recovery takes at least three months after decompressive surgery, with VA improving in 45 to 79% of patients. Predicting these improvements before the surgery is hard because the timing and amount of vision restoration can vary [2,8,20]. Many studies have noted that being younger, having symptoms for a shorter time, having smaller tumors, a higher MD value of preoperative VF, and a thicker peripapillary temporal RNFL thickness are important factors for better vision recovery following TSA-TR for pituitary tumors [21–24].

OCT is a reliable diagnostic tool for assessing RNFL and GCIPL thickness, providing objective and reproducible measurements of the thickness of RNFL and GCIPL. Bow-tie or horizontal band atrophy, seen as abnormal thinning in the nasal and temporal parts of the optic disc head, often occurs due to compression on the decussating nasal axonal fibers in the optic chiasm [9,25]. Thinning of the peripapillary RNFL, particularly in the nasal and temporal quadrants, is associated with horizontal band atrophy of optic disc [9]. OCT is widely recognized as a valuable clinical predictor of visual outcomes following surgical decompression for pituitary tumors that compress the optic chiasm. Patients who had a normal RNFL thickness exhibited a higher likelihood of visual recovery in contrast to those who had a thin RNFL. The thinning of RNFL on OCT indicates irreversible axonal damage, indicating a lower likelihood of visual function recovery after TSA-TR [9,26,27]. In this study, eyes with thick temporal RNFL thickness showed a greater improvement of MD in the visual field test, and the thick RNFL group had better postoperative visual field outcomes compared to the thin RNFL group. Furthermore, the thick RNFL group had a higher percentage of eyes with stable or improved postoperative MD changes than the thin RNFL group.

OCT can clearly demonstrates the structural loss of particular retinal layers, despite a near-normal appearance of the fundus. The thinning of macular of GCIPL can be detected in the earlier or subclinical stages of various optic neuropathy, which refers to primary axonal loss [16,17,27,28]. Balducci et al. [16] detected damage to the GCIPL starting six weeks before visual loss in Leber’s hereditary optic neuropathy (LHON). Changes in macular GC IPL thickness preceded changes in peripapillary RNFL thickness. The rates of reduction in mGCIPL and cpRNFL thicknesses were greatest between two to four weeks and four to six weeks after trauma [18]. Some studies reported that GCIPL was an independent risk factor of visual field defect recover after surgery [29,30]. In this study, there was a significant positive correlation between preoperative temporal RNFL thickness and the postoperative MD change rate, but there was no significant correlation between preoperative GCIPL thickness and postoperative MD change rate. Univariate analysis showed that preoperative RNFL thickness, GCIPL thickness, and MD value were predictors of favorable postoperative visual field outcomes, but multivariate analysis did not identify significant factors for predicting VF outcomes. Therefore, the GCIPL was not a good predictors of visual function outcomes following TSA-TR. Since GCIPL thickenss had already become significantly thinner at the time the RNFL thickness was reduces, it appears to have not been effective in predicting posoperative visual field outcomes.

Notably, this study has some limitations, including its retrospective design, limited number of participants, and lack of long-term follow-up to assess optic nerve recovery over time. However, the study’s strengths lie in the quantitative evaluation of OCT parameters and visual field tests, providing insights into prognostic indicators and comparing the effectiveness of RNFL and GCIPL measurements.

## Conclusions

In conclusion, this study suggests that patients with a thick RNFL have better postoperative MD values and a higher proportion of stable or improved postoperative MD changes compared to those with a thin RNFL. Eyes with thick preoperative temporal RNFL showed more noticeably an improvement of MD in VF. Specifically, preoperative thick RNFL could be a potential predictor of favorable visual field outcomes following TSA-TR, while macular GCIPL thickness does not appear to be a reliable predictor.
